# Genetic analysis of ancestry, admixture and selection in Bolivian and Totonac populations of the New World

**DOI:** 10.1186/1471-2156-13-39

**Published:** 2012-05-20

**Authors:** W Scott Watkins, Jinchuan Xing, Chad Huff, David J Witherspoon, Yuhua Zhang, Ugo A Perego, Scott R Woodward, Lynn B Jorde

**Affiliations:** 1 Department of Human Genetics, Eccles Institute of Human Genetics, University of Utah, 15 N 2030 E Rm 2100, Salt Lake City, UT, 84112, USA; 2 Department of Genetics and the Human Genetics Institute of New Jersey, Rutgers, The State University of New Jersey, 145 Bevier Rd, Piscataway, NJ, 08854, USA; 3 Dipartimento di Genetica e Microbiologia, Università di Pavia, Via Ferrata 1, 27100, Pavia, Italy; 4 Sorenson Molecular Genealogy Foundation, 2480 South Main Street, Suite 200, Salt Lake City, UT, 84115, USA

**Keywords:** Admixture, Ancestry Informative Markers (AIMs), Native Americans, Bolivian, Totonac, Positive selection

## Abstract

**Background:**

Populations of the Americas were founded by early migrants from Asia, and some have experienced recent genetic admixture. To better characterize the native and non-native ancestry components in populations from the Americas, we analyzed 815,377 autosomal SNPs, mitochondrial hypervariable segments I and II, and 36 Y-chromosome STRs from 24 Mesoamerican Totonacs and 23 South American Bolivians.

**Results and Conclusions:**

We analyzed common genomic regions from native Bolivian and Totonac populations to identify 324 highly predictive Native American ancestry informative markers (AIMs). As few as 40–50 of these AIMs perform nearly as well as large panels of random genome-wide SNPs for predicting and estimating Native American ancestry and admixture levels. These AIMs have greater New World vs. Old World specificity than previous AIMs sets. We identify highly-divergent New World SNPs that coincide with high-frequency haplotypes found at similar frequencies in all populations examined, including the HGDP Pima, Maya, Colombian, Karitiana, and Surui American populations. Some of these regions are potential candidates for positive selection. European admixture in the Bolivian sample is approximately 12%, though individual estimates range from 0–48%. We estimate that the admixture occurred ~360–384 years ago. Little evidence of European or African admixture was found in Totonac individuals. Bolivians with pre-Columbian mtDNA and Y-chromosome haplogroups had 5–30% autosomal European ancestry, demonstrating the limitations of Y-chromosome and mtDNA haplogroups and the need for autosomal ancestry informative markers for assessing ancestry in admixed populations.

## Background

The diaspora of humans into the New World is characterized mainly by prehistoric migrations from Asia at least 13,000 years ago [1] and by more recent migrations from Western Europe and Africa within the last 600 years [2]. A number of New World populations have remained isolated, while many others have experienced admixture from one or more Old World populations. These populations provide a unique opportunity for the analysis of genetic ancestry, admixture, and population structure.

Previous studies of mitochondrial genomes have shown that founding mitochondrial DNA (mtDNA) haplogroups from early migration event(s) are nested within northeastern and central Asian haplogroups (reviewed in reference [3]). Distinct geographic structuring of two Amerindian-specific subclades belonging to mtDNA haplogroups D and X has suggested that founding Paleo-Indian populations travelled both Pacific coastal and overland routes across Beringia 15,000–17,000 years ago [4]. There are several founding mtDNA lineages [5], but Native American Y-chromosome haplogroups appear limited to Q - M3 and C lineages [6]. Short tandem repeat (STR) variation in Amerindian Y-chromosome haplogroups suggests southwest Siberia as a plausible location for an ancestral New World founding population [7].

Only a few studies of indigenous American populations have been performed using large numbers of autosomal markers. Consistent with mitochondrial and Y-STR data, autosomal STR and SNP analyses support a southwestern Siberian / Central Asian origin for New World populations [8,9]. Genome-wide assays of STR markers show a clinal reduction of genetic diversity along a north – south axis across the Americas [8,10]. Several studies suggest that, despite large cultural and linguistic differences, many New World indigenous groups may be descendants of a single founding population [7,11,12]. Other studies have demonstrated substantial European and African ancestry in many populations of the Americas [13,14]. Recent admixture, founder effects, population bottlenecks [15] and selection can affect allele frequency and haplotype distributions, including disease-risk alleles. Admixture in some New World populations is also correlated with geographic distance, further confounding interpretations of early demographic events in the Americas [16]. Additional detailed studies of native and admixed populations using high-density autosomal markers are needed to resolve the effects of population history and to further characterize the genetic architecture of New World groups.

Here we perform a high-resolution genomic analysis of two previously uncharacterized New World populations with differing population histories using 815,377 autosomal SNPs, mtDNA sequence, Y-chromosome SNPs, and Y-chromosome STRs. We show that the Bolivians, but not Totonacs, have substantial European admixture. By comparing mitochondrial and Y-chromosome haplogroup ancestry estimates with estimates derived from autosomal data, we demonstrate the limitations of using only mtDNA and Y-chromosome data to predict an individual’s ancestry, especially in admixed populations. After removing admixed individuals, we identify autosomal SNPs that are highly differentiated between New and Old World populations. We produce a set of 324 ranked, New World-specific AIMS and show that some of the most highly differentiated SNPs coincide with high-frequency haplotypes common in native Bolivians, Totonacs, and five Native American populations from the Human Genome Diversity Project (HGDP).

## Results

**Figure 1:**
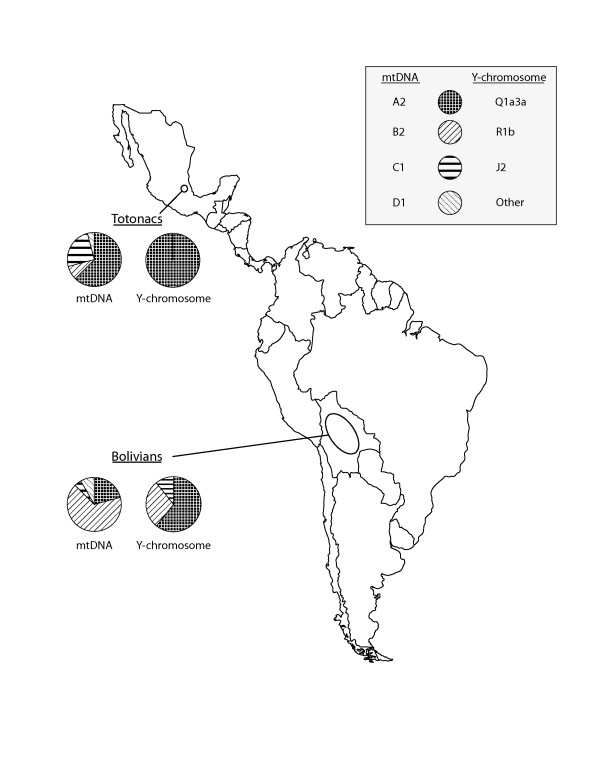
Sampling locations and the distribution of major mtDNA and Y-chromosome haplogroups for Mesoamerican Totonacs and South American Bolivians.

Ancestry for Mesoamerican and South American samples was assessed initially using mtDNA, and Y-STRs. We sought to identify samples with maximal New World and minimal European ancestry for additional high-throughput genotyping in a larger study of worldwide genetic variation [9]. The analysis of mtDNA HVS I and II showed that all Bolivian and Totonac samples belong to haplogroups A2, B2, C1 and D1, consistent with pre-Columbian New World maternal ancestry. Mitochondrial haplogroup A2 was the predominant lineage in the Totonacs (63%), while haplogroup B2 was prevalent in the Bolivians (71%). All Totonacs and 17 Bolivians (61%) had pre-Columbian Y-chromosomes (Q1a3a1). Consistent with historical accounts of male European admixture, 11 Bolivians (39%) carried Y-chromosome lineages that are common in Europe (R1b, J2, G) (Figure 1).

**Figure 2:**
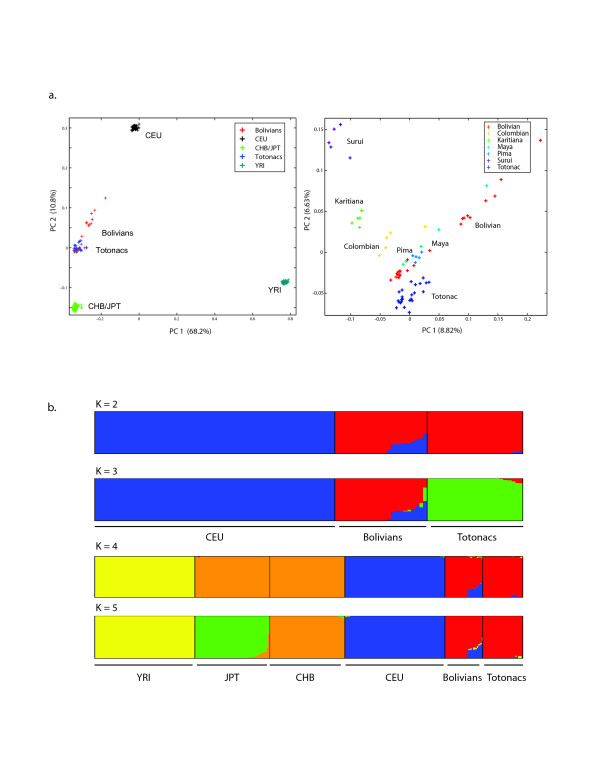
**a) Principal components plot of individual pairwise genetic distance estimates.** Panel 1 – most New World Totonac and Bolivian individuals are clustered and have smaller estimated distances to the HapMap CHB/JPT than to the CEU or YRI (~815 K SNPs). Panel 2 – data merged with five Native American HGDP populations typed on the Affymetrix 6.0 platform (~470 K SNPs). Each individual (+) is color coded by population. The percent variance accounted for by each principal component is indicated on the axes. **b)** Population structure analysis of Totonac and Bolivian individuals at *K* inferred ancestral populations using a genome-wide panel of 120,958 SNPs (r^2^ ≤ 0.2). Each individual is shown as a vertical bar with proportionate ancestry indicated by color. The top two panels show European attributable ancestry in ten Bolivians at *K* = 2, 3. The bottom two panels demonstrate greater similarity between the Totonacs and Bolivians than between other World populations (*K* = 4, 5), including CHB and JPT samples.

Totonac and Bolivian samples were genotyped on Affymetrix 6.0 microarrays. Following filtering (see Methods), a final autosomal dataset of 815,377 SNPs was assembled for the Totonacs (24), the Bolivians (23), and four HapMap populations (YRI, CEU, CHB, and JPT). Allele-sharing distances among individuals were estimated. A principal components analysis (PCA) of the individual distance estimates shows that most New World Bolivians and Totonacs are tightly clustered and more similar to eastern Asians than to Europeans (Figure 2a, panel 1). Nine Bolivians have substantially greater genetic affinity to HapMap Europeans than to other New World individuals based on their allele-sharing distances, suggesting European admixture in these samples. In the context of other southern Native Americans populations, the nine admixed Bolivians and one Mayan diverge from all other groups, while the Totonac are loosely clustered but relatively distinct from other samples (Figure2a, panel 2).

To estimate ancestry and the fraction of European admixture in each individual, we used the model-based population structure analysis implemented in the Admixture program [17]. The nine Bolivians identified as having potential European admixture by PCA show substantial European ancestry (22–47%) (Figure 2b). This analysis also detected one additional Bolivian with a small amount of European ancestry that was not clearly discerned by the PCA analysis. Inclusion of the HapMap African and East Asian populations in the population structure analysis yielded 2–8% potential African admixture in 8 Bolivians and 3 Totonacs. Though separated geographically by ~5,000 km, Bolivians and Totonacs remained identified by a single ancestry component (*K*) until *K* = 8 (not shown).

With the appropriate reference populations, high-density SNP data can be used to map the ancestry of chromosomal regions in admixed individuals. We constructed representative reference populations from the CEU samples and the non-admixed New World samples. Reference population genotypes were phased, and the Hapmix algorithm was used to estimate the probability that each SNP allele originated from one of the reference groups. This procedure was also performed for a randomly selected individual from each reference population. After optimizing parameters (see Methods), the average estimated fraction of European admixture in the 10 admixed Bolivians ranged from 0.13 to 0.48 (Table 1). These values were highly concordant with estimates from the population structure analysis performed using the Admixture algorithm (*r* = 0.99, *p* < 10^−5^).

**Table 1:** European admixture estimates for admixed Bolivian samples

	mtDNA haplogroup	Y-chromosome haplogroup	CEU - Fraction of genome^a^		
			Hapmix	Admixture (3 populations, K = 3)	Admixture (5 populations, K = 5)
*Admixed Bolivians*					
Bolivian 105	B2	R1b	0.48	0.47 ±0.02	0.41 ±0.02
Bolivian 869	A2	R1b	0.37	0.35 ±0.02	0.32 ±0.02
Bolivian 054	B2	J2	0.33	0.33 ±0.02	0.30 ±0.02
Bolivian 455	D1	Q1a3a1	0.30	0.28 ±0.02	0.24 ±0.02
Bolivian 853	B2	R1b	0.27	0.22 ±0.02	0.21 ±0.01
Bolivian 101	B2	R1b	0.25	0.23 ±0.02	0.21 ±0.01
Bolivian 817	B2	R1b	0.28	0.24 ±0.02	0.20 ±0.02
Bolivian 081	C1	Q1a3a1	0.26	0.23 ±0.02	0.23 ±0.02
Bolivian 458	B2	R1b	0.22	0.22 ±0.02	0.19 ±0.02
Bolivian 184	B2	Q1a3a1	0.13	0.05 ±0.02	0.09 ±0.02
*Non-admixed Controls*					
Totonac 867	A2	Q1a3a1	10^−5^	10^−5^ ±10^−16^	10^−5^ ±10^−16^
CEU NA11993	H	−	>0.99	>0.99 ±10^−16^	>0.99 ±0.10^−3^

^a^Estimated proportion of each genome attributable to European ancestry based on the CEU reference population and calculated from 815,377 SNPs (Hapmix) or 120,958 unlinked (r^2^ ≤ 0.2) SNPs (Admixture).

To better assess potential African admixture in the native Bolivians and Totonacs, we tested each population against the African YRI reference population using Hapmix. No African haplotype segments were found in the native Bolivians. Admixed Bolivian samples could not be tested against the African reference because the number of ancestry components exceeds two. The Totonacs yielded a total of 3 heterozygous YRI segments of less than 2.9 Mb found in two samples. This third approach suggests very minimal African admixture in the Totonacs or native Bolivian samples and excludes recent African admixture based on the small segment size.

The two New World populations provided us with an opportunity to compare ancestry predictions based on mtDNA, Y-chromosome, and autosomal data in non-admixed and admixed populations. The autosomal SNPs show that Totonacs have, at most, ~1.3% average admixture. All Totonac mtDNA and Y-chromosome haplogroups are consistent with pre-Columbian New World ancestry. In contrast, the Bolivians have, on average, ~12.1% admixture, attributable to 10 of the 23 individuals. Because five Bolivians with J or G Y-chromosome haplogroups were not typed on microarrays, our estimate of European autosomal admixture in the Bolivians is likely conservative due to this bias. Three of the ten admixed Bolivians carried pre-Columbian New World mtDNA and Y-chromosome haplogroups yet harbored ~5–30% autosomal European admixture at the individual level, demonstrating that ancestry prediction based on mtDNA and Y-chromosome haplogroups alone does not necessarily capture an individual’s actual ancestry.

**Figure 3:**
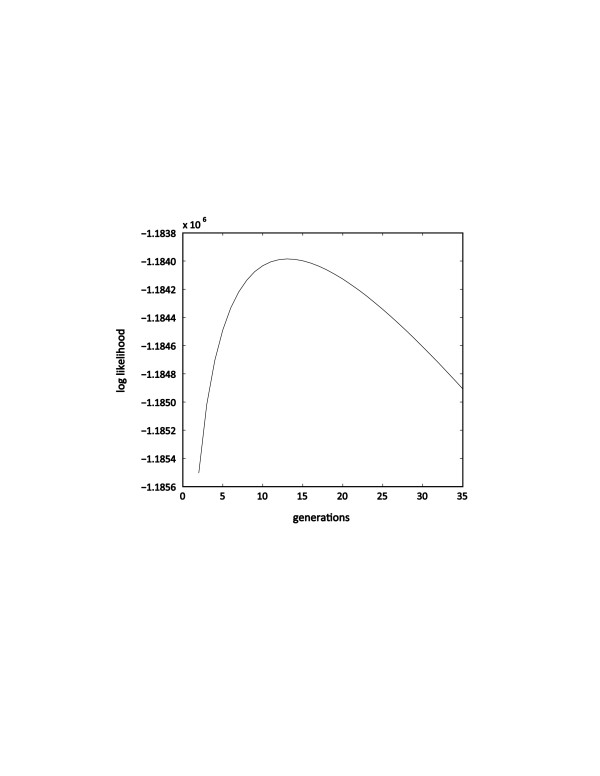
**Estimate for the age of admixture in Bolivians.** The Hapmix log likelihoods summed over all individuals and chromosomes is plotted for generations 2 through 35.

To estimate the average age of the admixture event, we calculated the likelihood of the data from each individual and chromosome under models that assumed different numbers of generations since admixture. The sum of likelihoods over all admixed individuals and chromosomes is maximized for a European admixture event 12 generations ago (Figure 3). This result suggests an approximate time of admixture of 360–384 years ago, assuming a generation time of 30–32 years [18].

We tested for familial relationships among the admixed Bolivians using a maximum-likelihood approach as implemented in the Estimation of Recent Shared Ancestry (ERSA) software package [19]. Only one of 45 pairwise comparisons among the ten admixed samples showed significant familial ties (*p* < 0.001; estimated at 9^th^ –degree relatives (6–19 degrees, 95% CI ), indicating that the admixture in the Bolivians is not explained by recent shared ancestry. Additionally, we used ERSA to test for relatedness in all Bolivian and Totonacs. Among Bolivians, we found 1 second-degree, 3 fourth-degree, 6 fifth-degree, and 10 sixth-degree relatives in 253 pairwise tests. Among Totonacs, there were 3 third-degree, 21 fourth-degree and 240 fifth-through seventh-degree relationships in 276 pairwise tests, typical of a population isolate with shared ancestry. Between Bolivian and Totonacs, no pairwise tests showed significant shared ancestry due to relatedness.

With the goal of producing a small set of AIMs that can rapidly identify indigenous American ancestry, we analyzed autosomal SNPs for New World ancestry information content in the non-admixed Bolivian and Totonac samples. SNPs were screened to identify those with low allele-frequency variance between the non-admixed Bolivians and Totonacs and high allele-frequency variance between the combined New World populations and each Old World population (YRI, CEU, CHB/JPT). A set of 324 AIMs was identified (see Methods and Additional file1: Table S1).

**Figure 4:**
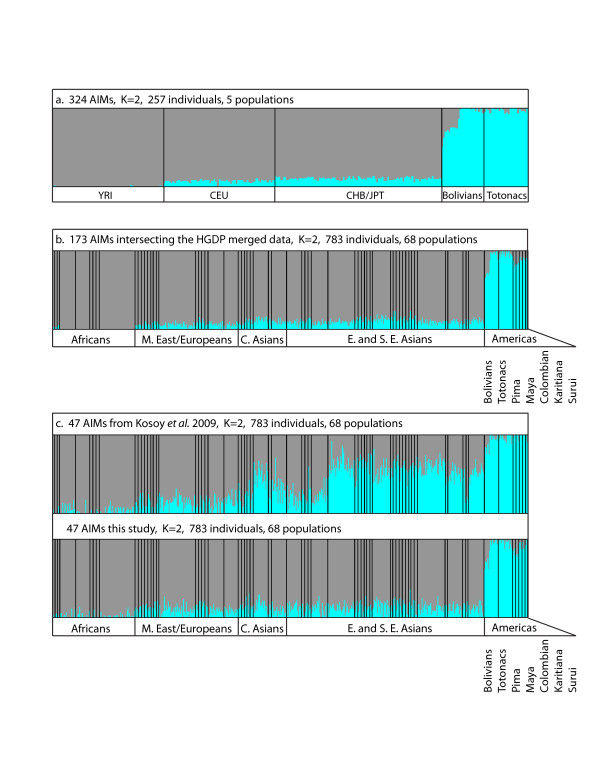
**Structure analysis of Bolivians and Totonacs using a panel of 324 AIMs.a)** New World ancestry is predicted for all Bolivians and Totonacs. A non-New World ancestry component is correctly distinguished in the ten Bolivians with European admixture. **b)** A subset of 173 AIMs present in the merged genome-wide data set (this study, [9] and [20]) identifies New World ancestry in other unrelated Native American populations and demonstrates transferability to other New World populations that were not used to ascertain the AIMs. **c)** 47 AIMs from Kosoy *et al.* present in the merged data.

The 324 markers accurately distinguished the Totonac and Bolivian samples from other populations in a population structure analysis (Figure 4a). No Old World sample exceeded 14% inferred New World ancestry, while all non-admixed Bolivian and Totonac samples had at least 91% inferred New World ancestry (median = 98%). The admixture estimates in the ten admixed Bolivian samples using these 324 AIMs were correlated with estimates from 120,958 unlinked genome-wide SNPs (*r* = 0.96, *p* < 0.001).

To assess the utility and portability of the AIMs to other New World populations and to compare these AIMs to other AIMs sets, we merged our data with samples from the Human Genome Diversity Project (HGDP) [20] which were typed on the Affymetrix platform. We also added worldwide populations examined previously by our group [9]. The five HGDP New World populations (Surui, Karitiana, Colombian, Maya, and Pima; *N* = 5 each), Bolivians, and Totonacs were assessed with all the AIMs present in both data sets (173 AIMs). These AIMs have power to distinguish all seven New World populations from 61 different Old World groups (Figure 4b). Kosoy *et al.* identified a set of 128 AIMs [21], and forty-seven of these AIMs were present in the merged data set. The 47 Kosoy AIMs identify Native American ancestry but do not separate the closely related Old World populations (central and eastern Asians) from the New World populations as effectively as an equal number of New World AIMs identified in this study (Figure 4c). The estimated fraction of non-New World ancestry for the larger panel remained well-correlated with a genome-wide estimates based on 130,288 unlinked SNPs (*r* = 0.95, *p* < 0.001). Thus, our panel of AIMs represents a small set of loci that efficiently identifies Native American ancestry in two unrelated ascertainment populations and five independent indigenous groups from Meso-and South America. We emphasize that our AIMs were designed for and are most effective for identifying New World ancestry under a two ancestry component model (K = 2).

**Figure 5:**
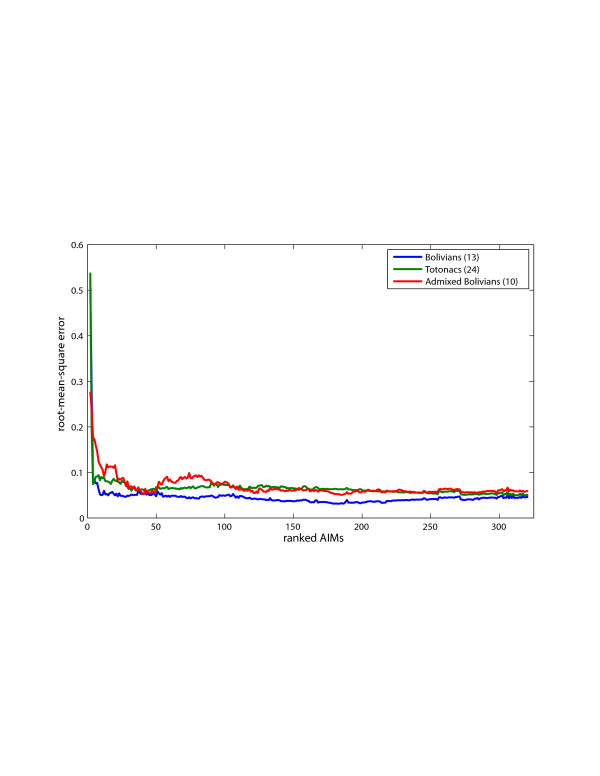
**Accuracy and performance of the 324 AIMs.** The root mean square error (RMSE) between Native American ancestry estimates using AIMs and the ancestry estimate using 120,598 genome-wide markers. (Note: the full AIMs panel (324 markers) produces ancestry estimates slightly lower than the genome-wide marker set, and thus the RMSE cannot achieve zero error with respect to the high-density genome-wide marker set.) AIMs are ordered from more informative (left) to less informative.

We assessed the minimum number of AIMs that could still effectively distinguish the Native American ancestry component in Totonacs, Bolivians, and admixed Bolivians. Using a resampling strategy, all 324 AIMs were ranked empirically for their ability to correctly estimate Native American ancestry in each of these populations as compared to the estimate from 120,958 genome-wide SNPs (see methods). The root-mean-squared error for these ranked sets of AIMs shows that the best 40–50 AIMs provide nearly the same accuracy for estimating Native American ancestry as all 324 AIMs (Figure 5). These estimates are within 7% of the genome-wide average and are conservative, under-estimating the actual proportion of Native American ancestry.

We next extended our SNP screening procedure to identify the most highly-differentiated New World SNPs in our data set. We selected SNPs comprising the upper 5% tails for standardized allele-frequency variance and Kullback–Leibler divergence of the derived allele for the New World (non-admixed Bolivians and Totonacs) versus each of the Old World groups. We obtained the intersection of the SNPs identified in these comparisons to find New World SNP alleles that were present in, but highly divergent from, the same alleles in each major Old World group, thus obtaining alleles with low variance in the Americas but high variance and high divergence between New and Old World groups. We found 22 SNPs in 17 genomic regions meeting these criteria (Table 2, Additional file 2: Table S2 and Additional file 3: Table S3).

**Table 2:** **Location and regional genomic features of highly-differentiated New World SN**Ps

SNP rs number	Chr	Position	Derived allele	XP-CLR Rank^a^ (percent)	XP-CLR score^b^	XP-EHH region	XP-EHH Rank^a^ (percent)	Gene	Gene function
rs2320170	2	95,603,500	A	10	7 / 7	−	−	5′ of TRIM43	Zn-finger protein
rs3774089	3	10,931,071	T	0.1	13 / 109	10,807,575 – 11,007,575	0.02	SLC6A11 (intronic)	GABA transporter
rs1344869	3	21,282,605	G	0.1	93/ 152	21,152,068 – 21,352,068	1	−	−
rs9847307	3	64,500,753	A	10	2 / 23	−	−	ADAMTS9 (intronic)	Protease
rs17617120	5	155,231,791	T	0.1	22 / 127	155,086,458 – 155,286,458	2	SGCD (intronic)	Cardiac, skeletal
rs17617422	5	155,249,830	G	0.1	80 / 130	155,086,458 – 155,286,458	2	SGCD (intronic)	Cardiac, skeletal
rs11960137	5	155,270,659	G	0.1	130 / 150	155,086,458 – 155,286,458	2	SGCD (intronic)	Cardiac, skeletal
rs2642515	7	145,998,474	T	1	103 / 103	145,884,552 – 146,084,552	2	CNTNAP2 (intronic)	Neurexin, regulated by FOXP2
rs174547	11	61,327,359	T	10	21 / 23	−	−	FADS1 (intronic)	Fatty acid desaturase
rs174548	11	61,327,924	C	10	20 / 25	−	−	FADS1 (intronic)	Fatty acid desaturase
rs174549	11	61,327,958	G	10	20 / 25	−	−	FADS1 (intronic)	Fatty acid desaturase
rs11610143	12	50,635,338	G	1	26 / 82	50,502,870 – 50,702,870	0.04	ACVR1B (intronic)	Signaling, growth factor receptor
rs7955663	12	127,800,083	A	10	1 / 11	−	−	−	−
rs1538142	13	37,344,432	C	0.1	123 / 177	37,244,768 – 37,444,768	2.5	5′ of TRPC4	Ca^2+^ channel
rs693092	13	87,858,156	G	1	24 / 68	87,851,770 – 88,051,770	2	−	−
rs9515075	13	88,033,482	C	0.1	105 / 189	87,851,770 – 88,051,770	2	−	−
rs566514	13	32,551,339	T	10	19 / 32	−	−	5′ of STARD13	GTP-binding, Lipid transfer
rs7170342	15	32,755,246	C	10	6 / 21	32,602,304 – 32,802,304	1	5′of AA496137	Expressed in testes
rs4924116	15	35,086,443	C	1	10 / 56	34,986,366 – 35,186,366	1	MEIS2 (intronic)	Homeobox, development
rs12439270	15	58,029,372	C	1	57 / 87	57,850,426 – 58,050,426	1	5′of FOXB1	Transcription factor
rs1452501	16	79,180,763	T	10	8 / 21	−	−	−	
rs470113	22	39,059,560	A	1	38 / 101	−	−	TNRC6B (3′UTR)	Nucleotide binding

Chr: chromosome; ^a^Ranking for a region based on the best score for the SNP ± 25 kb (XP-CLR) or ± 100 kb (XP-EHH), ranked empirically by percent of the distribution (*e.g.* the top 10%, 1%, 0.1%, …), comparisons to CHB/JPT; ^b^SNP score / Best score for region (SNP location ± 25 kb); positions based on hg18.

To evaluate the effects of selection and drift on the regions containing the highly differentiated alleles, we performed a genome-wide scan for selection in the New World samples using a multi-locus composite likelihood ratio test of allele-frequency differentiation as implemented in the XP-CLR program [22]. This method tests for alleles whose frequencies have changed more rapidly than predicted under a model of genetic drift and may be especially effective for detecting older selection signals. We used the combined non-admixed New World samples as the test population and the Old World Eurasians (CHB, JPT, and CEU) as the reference group. We also considered the CHB/JPT and the CEU as reference populations separately. Of the 22 SNPs identified as highly differentiated, 13 were included in the top 1% of the XP-CLR scan for selective sweeps, and the other 9 were in the top 10%, suggesting moderate effects of selection at these regions.

To control for the possibility that our highly-differentiated SNPs and the XP-CLR method are detecting similar signals based only on allele frequency differences between New and Old World populations, we used XP-EHH to identify candidate selection regions in the New World samples with extended haplotype homozygosity. To find candidate regions most likely to be specific to the Americas, we performed the XP-EHH test using the closely related CHB/JPT population as the reference group. Two SNPs identified by the highly-differentiated SNP screen occurred in genomic blocks that scored second and fifth in the XP-EHH test, and within these genomic blocks, the highest scoring XP-EHH SNP was located within 24 and 33 kb of the highly-differentiated SNPs, respectively. These high-scoring regions are contained with the solute carrier family 6 (*SLC6A11*) and the activin A type 1B (*ACVR1B*) receptor genes. An additional 11 highly-differentiated SNPs, from 9 independent regions, were located within genomic blocks that scored in the top 2.5% of the XP-EHH distribution (see Table 2).

## Discussion

The populations of the New World provide unique opportunities for the analysis of human demographic history, admixture, and disease. Many of these opportunities stem from 1) the genetic isolation of some New World groups from Old World populations, 2) a reduction in genetic diversity due to population bottlenecks, and 3) the recent introduction of distinct haplotypes and genetic diversity through admixture.

Our initial assessment of ancestry in the Totonac and Bolivian samples was performed using mtDNA and Y-chromosome haplogroups, a procedure commonly used to infer ancestry [23]. Only pre-Columbian mtDNA and Y-chromosome haplogroups were found in the Totonac population, and all Bolivian mtDNA haplogroups were also pre-Columbian in origin. Consistent with previous studies showing male-specific admixture in New World populations [24-26], some Bolivians had Y-chromosome haplogroups (J, G, R) common in European populations.

We assessed ancestry of the Bolivians using genome-wide autosomal markers and two different computational approaches. The ancestry estimates from two methods, Admixture and Hapmix, were highly correlated. Both methods showed that three Bolivians with pre-Columbian mtDNA and Y-chromosome haplogroups had ~5–30% European ancestry. Although ancestry for most samples could be correctly assigned using only mtDNA and Y-chromosome haplogroups, the finding illustrates the limitations of determining ancestry using only mtDNA and Y-chromosome haplogroups in admixed populations and is concordant with studies of admixture in other New World populations [13]. The average estimate of admixture in all Bolivians was ~12%. Although sampled as native Bolivians, the average likely reflects ancestry components of the non-admixed native Quechue/Aymara and the mixed ancestry mestizos. Our estimate of the age of admixture in the Bolivians is consistent with historical accounts of European admixture into the Americas. Due to constraints on genotyping and dispersed sampling, our study may underestimate the actual admixture and overestimate the timing of the European admixture.

Previous studies have provided excellent genome-wide panels of AIMs that are targeted to admixture mapping and ancestry identification applications [27,28]. Our study builds on the work performed by others [21,27], but uses an ascertainment approach to develop a marker set to separate Native American ancestry from non-Native American ancestry in a simple two ancestry component test. Comparing the Kosoy *et al.* set of 128 markers to our AIMs revealed only one overlapping marker and 7 other markers mapping within 100,000 bases of our markers. Our marker set had better ability to separate Native American ancestry from Eastern Asian ancestry for a matched number of markers, but a complete comparison could not be performed because of different initial SNP ascertainment sets. Our New World AIMs should also provide utility in combination with other more comprehensive world-wide AIMs sets to improve resolution for testing New World ancestry.

We obtained accurate separation of the New World groups from other populations with only 40–50 AIMs, but additional markers provided little increase in performance. Several explanations are possible including limited sample size, effects produced by our resampling procedure, stochastic effects caused by progressively adding less informative AIMs, or a combination of these factors.

Although costs for high-density genotyping arrays have steadily decreased, it is useful to perform very low-cost preliminary screening on a large number of samples. For instance, an initial screen of a large study cohort using only 40 highly informative AIMs should be sufficient to identify samples with optimal New World admixture proportions for admixture mapping prior to high-density microarray typing or genome sequencing. Using this two-stage approach, the need for expensive and time-consuming follow-up genotyping of candidate regions identified from standard admixture mapping panels can be reduced. Because our study included only Mesoamerican and South American groups, additional investigation will also be necessary to evaluate the accuracy of these AIMs in Native North American groups.

The most informative markers identified in our study were those with large frequency differences between New and Old World populations. Pickrell *et. al.* recently scanned for SNPs with large frequency differences between HGDP Yakuts and HGDP Mayans and identified rs12421620 in the dipeptidyl peptidase III (*DPP3*) gene as highly differentiated and a potential selection candidate in the New World [29]. We also identified rs12421620 as a member of the 324 AIMs set using non-admixed Bolivians and Totonacs.

At regions containing the most highly differentiated SNPs, haplotypes identical to those in the Bolivians and Totonacs were also found at high frequency in a limited sample of the HGDP Surui, Karitiana, Colombian, Maya, and Pima populations (see Additional file 3: Table S3). These five HGDP populations also have relatively little Old World admixture compared to many other New World groups (*e.g.* populations from Ecuador, the Dominican Republic, Mexico, Puerto Rico) [13]. Our findings support a large genetic contribution from a single founding group by showing that seven geographically separated Mesoamerican and South American populations all share identical high-frequency haplotypes in multiple regions of the genome. Further analyses are needed to determine whether strictly New World-specific polymorphisms are present on these haplotypes. Additionally, these haplotype regions should be examined in non-admixed Na-Dene and Eskimo-Aleut/Inuit groups to determine if these results can be replicated in northern North American populations.

We found that the most highly ancestry-differentiated SNPs in non-admixed Native Americans often coincided with regions having moderate selection signals as assessed by the XP-CLR metric. We anticipated a degree of overlap because both methods utilize allele-frequency differences between populations. The haplotypes in these regions are common in the non-admixed Bolivians, Totonacs, and New World HGDP populations examined and vary in length. Some of the haplotypes are relatively small, which suggests that selection in these regions occurred many generations ago and likely prior to the divergence of these groups. A brief period of strong selection on New World populations in the distant past would allow sufficient time for recombination to reduce the size of a selected haplotype, and XP-CLR is reported to detect older selection signals better than other linkage disequilibrium-based methods [22].

Evidence to further support some of these regions as selection candidates came from a cross-population screen for extended haplotype homozygosity. More than half of the 22 highly-differentiated SNP regions scored in the upper 2.5% of the XP-EHH distribution. The gamma-aminobutyric acid (GABA) transporter, *SLC6A11*, produced strong signals in all tests and is a candidate for additional studies. Nine other high-scoring XP-EHH regions have long haplotypes and are better candidates for recent positive selection than the regions with shorter haplotypes. Some of the selection signals seen are likely confounded with the strong recent population bottleneck in Native Americans, which should expedite fixation or loss of haplotype diversity in these populations. Additionally, the results of the XP-EHH and XP-CLR test were reference-population dependent. For instance, using a YRI reference group, regions in the top of the XP-EHH distribution for the Totonac and non-admixed Bolivians showed a high degree of overlap with New World selection candidates reported in other studies (e.g. KCNAB1) [29]. Thus, the evidence for selection candidates in Native Americans must be interpreted cautiously.

The populations of the Americas may provide new opportunities for the study of complex disease in two important ways. Population bottlenecks have led to a substantial reduction in genetic diversity among non-admixed populations of the New World. Lower allelic diversity and the absence of admixture in some New World populations may significantly reduce phenotypic variance for some traits, thus strengthening association signals between genotype and phenotype. Admixed populations of the New World also provide new opportunities to identify genetic components of complex disorders that have large differences in prevalence between populations. This approach is facilitated by identifying those New World groups and individuals with the optimal admixture proportions. The Totonac and Bolivian populations of Central and South America provide examples of groups amenable to each approach.

## Conclusions

The genetic structure of some native Bolivians has been substantially influenced by admixture from Europeans, which we estimate to have occurred approximately 360–384 years ago. Consistent with historical accounts of male admixture, Y-chromosome haplogroups typical of Europeans were found in 39% of our Bolivian samples. No evidence of African admixture was found in native Bolivians. The Mesoamerican Totonacs have little evidence of European or African admixture. Our analysis indicates that some admixed Bolivians have Native American mtDNA and Y-chromosomes but harbor up to 30% European autosomal ancestry, demonstrating the need for autosomal markers to assess ancestry in admixed populations.

From a dense genome-wide panel of 815,377 markers, we developed a set of 324 AIMs, specific for Native American ancestry. As few a 40–50 of these markers successfully predict New World ancestry in the ascertainment panel of Bolivians and Totonacs. The markers easily distinguish New World from Old World ancestry, even for populations more closely related to the Americas such as central and eastern Asians, and were effective for New World vs. Old World comparisons in five other geographically and culturally distinct populations of the Americas. SNPs demonstrating very high divergence between the two Native American populations and major Old World populations are found on haplotypes that are shared and occur at similar frequencies in other indigenous low-admixture American populations examined here (*i.e.* Pima, Maya, Colombian, Karitiana, and Surui). After excluding the possibility of recent relatedness, our results indicate that native Bolivians and Totonacs share ancestry with other American populations through a substantial contribution from a common founding population, population bottlenecks, and possible natural selection on functional variation.

## Methods

Mesoamerican Totonacs (24) were sampled from an isolated rural location near Filomeno Mata, Veracruz, in southern Mexico. South American Bolivians (28) were obtained from several locations in Bolivia. All subjects were collected as unrelated samples, and all subjects’ grandparents originated from the same geographic region. All samples were collected with informed consent by the Sorenson Molecular Genealogical Foundation (SMGF) as part of a worldwide sample collection project. The study was approved by the Western Institutional Review Board.

Approximately 2 ml of saliva were obtained from each individual using a mouthwash kit. Sample DNA was extracted using a standard alkaline-SDS procedure. Mitochondrial hypervariable segments (HVS) I and II from nucleotide position 16,024 through 576 were determined by Sanger sequencing. Along with basal mtDNA clade variation, pre-Columbian mtDNA lineages were inferred with the following key variants: Haplogroup A: A – 16290 T, 16319A, 235 G; A2 – 16111 T, 146 C, 153 G; Haplogroup B: B – 16189 C; B4 – 16217 C; B4b – 499A, B2 – 16136 T, [16183d]; Haplogroup C: C – 16298 C, 16327 T, 249d; C1 – 16325 C, 290-290d; C1b – 493 G; C1d – 16051 G; Haplogroup D: D – 16362 C; D1 – 16325 C. Haplogroup X was not observed. To assign Y-chromosome lineages, samples were genotyped for 36 Y-chromosome STR loci: DYS385, DYS388, DYS389I, DYS389B, DYS390, DYS391, DYS392, DYS393, DYS394, DYS426, DYS437, DYS438, DYS439, DYS441, DYS444, DYS445, DYS446, DYS447, DYS448, DYS449, DYS452, DYS454, DYS455, DYS456, DYS458, DYS459, DYS460, DYS461, DYS462, DYS463, DYS464, GGAAT1B07, YCAII, YGATAA10, YGATAC4, and YGATAH4. The Bolivians were typed for 11 additional Y-SNPs: M172, M173, SRY10831.2, M124, M122, M3, M74, M9, M20, M216, and M89. Y-chromosome lineages were assigned probabilistically using 35 (of the 36) STR loci [30]. Haplogroups for the Bolivians were verified or further resolved with the 11 additional Y-chromosome SNPs. All Totonac lineages were verified with Y-chromosome SNPs M242 and M3.

Autosomal SNP data were generated using Affymetrix 6.0 microarrays. Three Bolivians with European Y-haplogroups (G and J) were removed prior to microarray genotyping. Two-hundred thirteen SNPs showing strong deviation (*p* < 5.5 x10^−8^) from Hardy-Weinberg expectations were removed as previously described [9]. Pairwise genetic distances were estimated as the average fraction of alleles shared between two individuals over all loci. Two pairs of Bolivians had allele sharing genetic distances of < 0.13, suggesting relatedness [9]. One sample from each of these pairs was removed, yielding 23 Bolivian samples for analysis. The identity-by-descent haplotype-sharing analysis was performed using the ERSA software [19]. Although many New World HGDP samples show substantial relatedness, the HGDP samples used here were not inferred to be close relatives in a previous study [31]. Affymetrix 6.0 genotypes for the 210 unrelated HapMap samples were obtained from the HapMap project website, and the same SNP selection criteria were applied to HapMap samples. The filtered HapMap dataset was combined with the dataset generated in this study to assemble a final data set of 815,377 autosomal SNPs for Totonacs (24), Bolivians (23), unrelated HapMap Yoruba (YRI) (60), unrelated HapMap CEPH (CEU) (60), HapMap Han Chinese (CHB) (45), and HapMap Japanese (JPT) (45). Principal components analysis was performed on pairwise allele-sharing distances using the princomp program and plotted with graphics tools provided in the Matlab software package (Mathworks, USA).

Genome-wide admixture estimates and their standard errors were obtained with the Admixture algorithm (version 1.02) [17] after pruning the data for SNPs with pairwise r^2^ ≥ 0.2. Runs at an r^2^ pruning of 0.5, or no pruning, produced similar results. We performed the Admixture analysis to determine which Bolivian samples were admixed and demonstrated that there were two major ancestry components in a subset of Bolivians. We then used the Hapmix program, which is limited to two population comparisons (K = 2), to analyze admixture in the Bolivians. Genome-wide SNPs were assembled for a CEU reference population (60 individuals) and a New World reference population (24 Totonacs plus 13 non-admixed Bolivian individuals). SNP data for each reference population were phased with imputation of missing data using the Beagle software package [32]. Unphased genotypes for all SNPs were assembled for the potentially admixed Bolivian samples. The admixed chromosomes were phased and reconstructed with probability estimates of European (CEU) ancestry using the Hapmix program [33]. Most Hapmix run parameters were set using guidelines as suggested by the authors. Because New World populations have much smaller effective population sizes (N_*e*_) than Europeans [15], the New World recombination parameter, ρ_2_, was scaled (0.15) relative to the CEU parameter, ρ_1_. Final runs were performed for each individual and each chromosome, varying the number of generations since admixture (*n* = 2, 3 … 35). The time of admixture was estimated by computing the likelihood of the data from all chromosomes and all individuals over a range of generations since the admixture event and selecting the value that maximized the summed likelihoods. Individual genome-wide estimates of admixture were calculated as the average expected probability of the number of CEU copies over all SNPs.

To identify ancestry informative markers, each of the 815,377 markers was assessed for ancestry information content between the New World and HapMap groups using standardized allelic variance (*f*_*d*_*)*[34], calculated as *f*_*d*_ = (*p*_*a*_ – *p*_*b*_)^2^ /[4*p*_*ab*_(1-*p*_*ab*_)], where *p*_*a*_ and *p*_*b*_ are the derived allele frequencies in population *a*, population *b*, respectively, and *p*_*ab*_ is the average derived allele frequency in populations *a* and *b*. A threshold of *f*_*d*_ ≤ 0.1 was used to screen for markers with low population differentiation between the Totonacs and non-admixed Bolivians. A threshold of *f*_*d*_ ≥ 0.3 was used to screen for markers with high variance between a combined Totonac + non-admixed Bolivian population and each Old World population (YRI, CEU, or CHB + JPT). SNPs common to all three New vs. Old World screens were retained (845 markers). This AIMs set was further reduced to 324 AIMs markers by removing 1) one of every pair of SNPs with pairwise r^2^ exceeding 0.2 in a 100-SNP sliding window advanced by 10 SNPs and 2) all SNPs within 100 kb of one another. To obtain the highly divergent SNP set, we repeated this process but set the minimum value of *f*_*d*_ as the 5% tail for each distribution (range 0.3085 to 0.5804, all markers retained). We then required the SNP to be in the upper 5% tail of the Kullback–Leibler divergence (*D*) for the derived allele *i*, where $D=\sum_{i=1}^{N}\;p1_{i}log\;\frac{p1_{i}}{p2_{i}}+p2_{i}log\;\frac{p2_{i}}{p1_{i}}$ and *p*1_i_ and *p*2_i_ are the frequencies of allele *i* in populations 1 and 2 [35,36]. We note that the variance and divergence measures are correlated (*r* = 0.696) but have different distributions. AIMs passing the screening process were checked against HapMap and dbSNP for frequency and strand assignment. Seven highly-differentiated G/C and A/T AIMs were removed due to the possibility of strand assignment confounding.

We empirically determined the ranking of the 324 AIMs by resampling. Subsets of 50 AIMs were randomly selected without replacement from the 324 AIMs. Using the average Native American ancestry estimate from 120,958 genome-wide SNPs as the true ancestry fraction, we iteratively screened for sets of AIMs producing average Native American ancestry component estimates within 10% of the genome-wide average estimate at K = 5 populations and retained 10,000 sets. The AIMs were ranked by total number of times each AIM was seen over all retained sets. Totonacs and non-admixed Bolivians were analyzed independently. The sum of the ranks in the two populations was used to determine the final ranking for each AIM. To assess the minimum number of AIMs need to estimate ancestry, we calculated admixture estimates for Totonacs, non-admixed Bolivians, and admixed Bolivians using sets of 2 to 324 AIMs ranked from most to least informative as described above, and calculated the root mean squared error for each set.

Selection scans were performed using XP-CLR and XP-EHH [22,37]. For XP-CLR, the New World populations (Totonac and non-admixed Bolivians) were analyzed against a reference population of Eurasians (CEU, CHB, and JPT). XP-CLR is less influenced by SNP ascertainment bias, a known issue with most SNP microarrays [38,39], and may detect older selection events better than linkage disequilibrium based methods. XP-CLR scans were performed on Beagle-phased haplotypes using a 0.5 cM sliding window and 2 kb grid setting with a maximum of 100 SNPs per window. The XP-EHH analysis was performed using the combined Totonac and non-admixed Bolivians as the test population against the CHB/JPT, CEU, and YRI reference populations. Genomic regions, in 200 kb blocks, were ordered based on the highest scoring SNP in the block and rank determined empirically from the distribution.

## Competing interests

The authors declare no competing financial interests.

## Authors’ contributions

WSW designed the study, performed genotyping, analyzed the data, and drafted the manuscript. JX designed the study and edited the manuscript. CH performed ERSA analysis of the admixed individuals. DJW provided statistical consultation and edited the manuscript. YZ performed genotyping. UAP collected the samples and analyzed the mtDNA haplogroups. SRW collected the samples. LBJ edited the manuscript, provided sponsorship, funding, and laboratory facilities. All authors read and approved the final manuscript.

## Supplementary Material

Additional file 1: Table S1. 324 ranked Native American AIMs. Click here for file

Additional file 2: Table S2. Highly-differentiated SNP frequencies. Click here for file

Additional file 3: Table S3. Haplotypes and haplotype frequencies associated with the highly-differentiated SNPs. Genotype data and Affymetrix cel files for the Totonac and Bolivian samples can be downloaded from the Gene Expression Omnibus (GEO) archive (GSE29851). Click here for file
