# Supplementary material for: Genetic analysis of ancestry, admixture and selection in Bolivian and Totonac populations of the New World
Source: BMC Genet. 2012 May 20;13:39. doi: 10.1186/1471-2156-13-39 (PMC3432609; doi:10.1186/1471-2156-13-39)
Supplement: Additional file 1: Table S1. — 324 ranked Native American AIMs. [file 1471-2156-13-39-S1.docx]

Supplemental Table 1. 324 Ranked Native American AIMs

| Rs number | Chr | Rank | Position (hg18) | Allele | Population frequency | | | |
| --- | --- | --- | --- | --- | --- | --- | --- | --- |
|  |  |  |  |  | YRI | CEU | CHB/JPT | New World* |
| rs12403612 | 1 | 139 | 4911150 | G | 0.08 | 0.10 | 0.01 | 0.67 |
| rs2898917 | 1 | 212 | 6909928 | T | 0.01 | 0.06 | 0.04 | 0.68 |
| rs920601 | 1 | 12 | 29994884 | G | 0.27 | 0.40 | 0.32 | 0.96 |
| rs530373 | 1 | 31 | 43842619 | A | 0.24 | 0.30 | 0.41 | 0.96 |
| rs17110221 | 1 | 312 | 54518055 | C^§^ | 0.09 | 0.07 | 0.14 | 0.69 |
| rs12563251 | 1 | 216 | 54679755 | T | 0.06 | 0.13 | 0.23 | 0.78 |
| rs1342514 | 1 | 221 | 56959948 | G^§^ | 0.25 | 0.31 | 0.23 | 0.86 |
| rs3790857 | 1 | 131 | 63884454 | T | 0.09 | 0.13 | 0.14 | 0.70 |
| rs4650224 | 1 | 100 | 73939547 | A | 0.02 | 0.19 | 0.28 | 0.83 |
| rs12407548 | 1 | 112 | 104533350 | G | 0.16 | 0.08 | 0.21 | 0.77 |
| rs2296380 | 1 | 254 | 111522877 | T | 0.29 | 0.10 | 0.21 | 0.86 |
| rs7513936 | 1 | 83 | 111726430 | A | 0.21 | 0.40 | 0.32 | 0.93 |
| rs12405235 | 1 | 58 | 151412368 | A | 0.93 | 1.00 | 0.96 | 0.39 |
| rs4656517 | 1 | 57 | 165122683 | G | 0.22 | 0.23 | 0.18 | 0.84 |
| rs6427063 | 1 | 184 | 165343158 | T | 0.00 | 0.22 | 0.06 | 0.77 |
| rs7555140 | 1 | 304 | 185184143 | A | 0.90 | 0.68 | 0.85 | 0.10 |
| rs10921053 | 1 | 203 | 190234692 | G | 0.01 | 0.26 | 0.25 | 0.85 |
| rs568 | 1 | 82 | 201979756 | G | 0.79 | 0.85 | 0.84 | 0.18 |
| rs16855698 | 1 | 96 | 203749747 | C | 0.08 | 0.04 | 0.22 | 0.90 |
| rs10910204 | 1 | 265 | 231719647 | C | 0.93 | 0.81 | 0.77 | 0.19 |
| rs10926111 | 1 | 8 | 238293444 | C | 0.48 | 0.48 | 0.41 | 0.97 |
| rs13018508 | 2 | 311 | 16258081 | T | 0.01 | 0.06 | 0.00 | 0.61 |
| rs12474314 | 2 | 144 | 23841103 | G^§^ | 0.00 | 0.00 | 0.05 | 0.59 |
| rs10173959 | 2 | 244 | 30882449 | T | 0.10 | 0.23 | 0.22 | 0.80 |
| rs4952586 | 2 | 40 | 42123256 | A | 0.79 | 0.85 | 0.63 | 0.09 |
| rs10490718 | 2 | 22 | 67597762 | T | 0.13 | 0.35 | 0.41 | 0.97 |
| rs10496176 | 2 | 62 | 70027133 | C | 0.19 | 0.17 | 0.34 | 0.91 |
| rs6724915 | 2 | 313 | 71916980 | T | 0.12 | 0.13 | 0.13 | 0.72 |
| rs1400681 | 2 | 134 | 72124459 | C | 0.06 | 0.09 | 0.02 | 0.63 |
| rs11682182 | 2 | 291 | 95741937 | T | 0.88 | 0.68 | 0.68 | 0.10 |
| rs6732839 | 2 | 276 | 100257835 | C | 0.99 | 0.98 | 0.99 | 0.50 |
| rs4624401 | 2 | 108 | 100646869 | T^§^ | 0.03 | 0.21 | 0.07 | 0.91 |
| rs10206927 | 2 | 315 | 100834319 | G^§^ | 0.97 | 0.78 | 0.79 | 0.18 |
| rs6752589 | 2 | 292 | 102221730 | A | 0.12 | 0.21 | 0.21 | 0.77 |
| rs1402070 | 2 | 236 | 156419907 | G | 0.03 | 0.20 | 0.19 | 0.76 |
| rs17266406 | 2 | 272 | 157132783 | C | 0.00 | 0.03 | 0.00 | 0.69 |
| rs10174247 | 2 | 113 | 159781041 | A | 0.08 | 0.36 | 0.34 | 0.91 |
| rs12465777 | 2 | 252 | 160787137 | A^§^ | 0.00 | 0.07 | 0.02 | 0.66 |
| rs734993 | 2 | 307 | 173734245 | C | 0.08 | 0.20 | 0.13 | 0.79 |
| rs3754953 | 2 | 300 | 179326264 | A | 0.88 | 0.93 | 0.78 | 0.21 |
| rs8179655 | 2 | 117 | 180558325 | G | 0.84 | 0.76 | 0.76 | 0.11 |
| rs4233776 | 2 | 268 | 181427026 | A | 0.10 | 0.07 | 0.17 | 0.74 |
| rs2696115 | 2 | 158 | 183236915 | T | 0.18 | 0.28 | 0.29 | 0.88 |
| rs1108939 | 2 | 274 | 190942435 | A | 0.05 | 0.17 | 0.19 | 0.81 |
| rs4675938 | 2 | 257 | 242126050 | T | 0.00 | 0.00 | 0.16 | 0.78 |
| rs1153539 | 3 | 153 | 2224261 | G | 0.04 | 0.28 | 0.11 | 0.85 |
| rs3774089 | 3 | 213 | 10931071 | T | 0.01 | 0.11 | 0.17 | 0.87 |
| rs1018183 | 3 | 200 | 11954988 | G | 0.16 | 0.23 | 0.25 | 0.80 |
| rs6780694 | 3 | 199 | 21364344 | C | 0.10 | 0.01 | 0.34 | 0.91 |
| rs17010014 | 3 | 67 | 21911046 | C | 0.07 | 0.00 | 0.14 | 0.73 |
| rs7430140 | 3 | 65 | 23197513 | C | 0.02 | 0.24 | 0.28 | 0.84 |
| rs9843698 | 3 | 103 | 24265749 | C | 0.00 | 0.13 | 0.07 | 0.75 |
| rs9827642 | 3 | 64 | 24932021 | A | 0.17 | 0.38 | 0.24 | 0.91 |
| rs795448 | 3 | 215 | 30775624 | G | 0.04 | 0.18 | 0.14 | 0.80 |
| rs4678506 | 3 | 161 | 33236439 | A | 0.23 | 0.08 | 0.24 | 0.82 |
| rs3772170 | 3 | 68 | 43603851 | A | 0.25 | 0.04 | 0.10 | 0.84 |
| rs2306569 | 3 | 271 | 53249642 | C | 0.07 | 0.33 | 0.32 | 0.86 |
| rs1179911 | 3 | 9 | 54870962 | T | 0.53 | 0.61 | 0.53 | 0.01 |
| rs358012 | 3 | 287 | 55100681 | T | 0.16 | 0.16 | 0.27 | 0.82 |
| rs17062824 | 3 | 75 | 60326601 | G^§^ | 0.00 | 0.08 | 0.22 | 0.80 |
| rs4317089 | 3 | 226 | 64499600 | T | 0.01 | 0.01 | 0.22 | 0.77 |
| rs4856894 | 3 | 162 | 67915983 | T | 0.00 | 0.07 | 0.08 | 0.76 |
| rs17016013 | 3 | 168 | 78510541 | C | 0.00 | 0.01 | 0.14 | 0.76 |
| rs2062430 | 3 | 142 | 85177771 | G | 0.12 | 0.08 | 0.25 | 0.80 |
| rs16838563 | 3 | 163 | 98501169 | T | 0.05 | 0.02 | 0.06 | 0.61 |
| rs4687862 | 3 | 93 | 118970261 | T | 0.22 | 0.23 | 0.26 | 0.81 |
| rs7614907 | 3 | 172 | 120559179 | T | 0.07 | 0.06 | 0.00 | 0.58 |
| rs4676738 | 3 | 245 | 122811490 | G | 0.07 | 0.17 | 0.06 | 0.76 |
| rs6785802 | 3 | 256 | 136770617 | C | 0.93 | 0.98 | 0.98 | 0.42 |
| rs1500864 | 3 | 114 | 148501445 | T | 0.03 | 0.24 | 0.39 | 0.95 |
| rs7627166 | 3 | 107 | 156496887 | A | 0.99 | 1.00 | 1.00 | 0.38 |
| rs13065060 | 3 | 148 | 169404190 | G^§^ | 0.18 | 0.26 | 0.26 | 0.82 |
| rs2946399 | 4 | 278 | 23522677 | T | 0.00 | 0.13 | 0.24 | 0.85 |
| rs10866391 | 4 | 228 | 31740080 | C^§^ | 0.89 | 0.92 | 0.84 | 0.26 |
| rs1437630 | 4 | 118 | 36401829 | C | 0.00 | 0.12 | 0.22 | 0.77 |
| rs12499585 | 4 | 253 | 40387644 | T | 0.00 | 0.11 | 0.31 | 0.87 |
| rs1355806 | 4 | 165 | 69674823 | C | 0.04 | 0.14 | 0.13 | 0.69 |
| rs1991948 | 4 | 104 | 73473800 | C | 0.11 | 0.03 | 0.04 | 0.69 |
| rs11733293 | 4 | 219 | 103764301 | C | 0.77 | 0.65 | 0.63 | 0.09 |
| rs6533470 | 4 | 76 | 111000760 | T | 0.63 | 0.65 | 0.65 | 0.09 |
| rs12500301 | 4 | 137 | 117939378 | G | 1.00 | 0.97 | 1.00 | 0.47 |
| rs6833309 | 4 | 124 | 155785421 | A | 0.13 | 0.05 | 0.10 | 0.72 |
| rs1991727 | 4 | 20 | 174399744 | T | 0.42 | 0.28 | 0.26 | 0.95 |
| rs1481121 | 4 | 16 | 174584972 | C | 0.25 | 0.47 | 0.35 | 0.97 |
| rs17694007 | 5 | 217 | 4787545 | A | 0.00 | 0.06 | 0.02 | 0.61 |
| rs6554588 | 5 | 81 | 10654148 | T | 0.35 | 0.24 | 0.24 | 0.90 |
| rs7732720 | 5 | 210 | 11189164 | A | 0.03 | 0.08 | 0.06 | 0.64 |
| rs1446040 | 5 | 27 | 15177021 | A | 0.01 | 0.36 | 0.28 | 0.96 |
| rs12521185 | 5 | 123 | 15592360 | A | 0.00 | 0.18 | 0.08 | 0.74 |
| rs1867723 | 5 | 86 | 16550102 | T | 0.03 | 0.28 | 0.42 | 0.95 |
| rs11738873 | 5 | 140 | 22467734 | T | 0.03 | 0.20 | 0.24 | 0.82 |
| rs1366363 | 5 | 242 | 29985377 | A | 0.02 | 0.05 | 0.16 | 0.76 |
| rs3805490 | 5 | 262 | 40827910 | T | 0.98 | 0.78 | 0.78 | 0.20 |
| rs3797250 | 5 | 205 | 53538991 | C | 0.11 | 0.23 | 0.12 | 0.80 |
| rs1286371 | 5 | 110 | 68029954 | C | 0.07 | 0.31 | 0.23 | 0.92 |
| rs12657828 | 5 | 183 | 79121482 | G | 0.32 | 0.13 | 0.22 | 0.88 |
| rs10942426 | 5 | 246 | 85406603 | C | 0.07 | 0.09 | 0.15 | 0.75 |
| rs17135515 | 5 | 109 | 112626846 | C | 0.20 | 0.05 | 0.06 | 0.77 |
| rs3891925 | 5 | 166 | 113113476 | T | 0.01 | 0.13 | 0.04 | 0.72 |
| rs17462291 | 5 | 152 | 120878259 | G | 0.01 | 0.13 | 0.14 | 0.81 |
| rs4527598 | 5 | 41 | 124253166 | C | 0.33 | 0.40 | 0.43 | 0.96 |
| rs331082 | 5 | 60 | 127796500 | T | 0.16 | 0.10 | 0.05 | 0.81 |
| rs4705021 | 5 | 171 | 146534624 | A | 0.28 | 0.35 | 0.26 | 0.89 |
| rs17703748 | 5 | 284 | 147249909 | A | 0.00 | 0.04 | 0.06 | 0.59 |
| rs4147470 | 5 | 24 | 148508300 | T | 0.24 | 0.50 | 0.44 | 0.99 |
| rs7379703 | 5 | 316 | 149005182 | T^§^ | 0.05 | 0.20 | 0.18 | 0.76 |
| rs1422363 | 5 | 3 | 151826429 | T | 0.23 | 0.33 | 0.26 | 0.97 |
| rs11951160 | 5 | 248 | 154557485 | G | 0.07 | 0.14 | 0.29 | 0.84 |
| rs17617120 | 5 | 89 | 155231791 | T | 0.06 | 0.12 | 0.19 | 0.89 |
| rs2272600 | 5 | 182 | 161501518 | A | 1.00 | 0.88 | 0.82 | 0.24 |
| rs17732485 | 5 | 225 | 168027033 | C | 0.00 | 0.00 | 0.08 | 0.60 |
| rs10063658 | 5 | 66 | 169063925 | T | 0.25 | 0.03 | 0.16 | 0.85 |
| rs414247 | 6 | 50 | 6117419 | G | 0.98 | 0.60 | 0.61 | 0.06 |
| rs11758829 | 6 | 232 | 10193421 | C^§^ | 0.00 | 0.12 | 0.08 | 0.69 |
| rs12526712 | 6 | 155 | 10543206 | G | 0.01 | 0.17 | 0.28 | 0.83 |
| rs1736502 | 6 | 267 | 13362639 | G | 0.03 | 0.06 | 0.07 | 0.60 |
| rs12529358 | 6 | 63 | 19535623 | C | 0.05 | 0.23 | 0.14 | 0.82 |
| rs2894311 | 6 | 285 | 33076317 | C | 0.00 | 0.08 | 0.06 | 0.64 |
| rs4713975 | 6 | 97 | 36503739 | A | 0.14 | 0.07 | 0.13 | 0.69 |
| rs6941539 | 6 | 111 | 41827088 | T | 0.03 | 0.03 | 0.14 | 0.78 |
| rs17747449 | 6 | 229 | 51910225 | T | 0.06 | 0.04 | 0.00 | 0.59 |
| rs12665578 | 6 | 156 | 52413500 | T | 0.06 | 0.18 | 0.19 | 0.74 |
| rs9352947 | 6 | 85 | 81886762 | C^¥^ | 1.00 | 1.00 | 1.00 | 0.47 |
| rs9688767 | 6 | 125 | 83638977 | C | 0.24 | 0.10 | 0.13 | 0.80 |
| rs4945745 | 6 | 106 | 106736406 | A^§^ | 0.00 | 0.09 | 0.14 | 0.69 |
| rs12526640 | 6 | 214 | 121442680 | G | 0.00 | 0.06 | 0.01 | 0.57 |
| rs933239 | 6 | 231 | 122190619 | G | 0.00 | 0.12 | 0.02 | 0.78 |
| rs2816140 | 6 | 6 | 122543916 | C | 0.40 | 0.23 | 0.36 | 0.93 |
| rs17074221 | 6 | 269 | 145164810 | G^§^ | 0.21 | 0.08 | 0.25 | 0.82 |
| rs3861453 | 6 | 243 | 148365023 | T | 0.17 | 0.15 | 0.19 | 0.76 |
| rs2057557 | 6 | 84 | 150536566 | A | 0.11 | 0.21 | 0.20 | 0.89 |
| rs1124264 | 6 | 167 | 151657419 | G | 0.04 | 0.10 | 0.25 | 0.82 |
| rs6557339 | 6 | 115 | 154569846 | C | 0.21 | 0.26 | 0.06 | 0.85 |
| rs1510226 | 6 | 188 | 160736399 | C | 0.00 | 0.00 | 0.05 | 0.58 |
| rs16892196 | 6 | 149 | 161478088 | A | 0.01 | 0.13 | 0.17 | 0.73 |
| rs4709836 | 6 | 98 | 164636380 | T | 0.10 | 0.20 | 0.29 | 0.84 |
| rs4720322 | 7 | 196 | 4542106 | T | 0.23 | 0.24 | 0.23 | 0.85 |
| rs4518565 | 7 | 189 | 5205121 | G | 0.07 | 0.26 | 0.21 | 0.90 |
| rs9648583 | 7 | 227 | 5877817 | A | 0.08 | 0.21 | 0.08 | 0.78 |
| rs2080161 | 7 | 120 | 13297675 | C | 0.03 | 0.21 | 0.32 | 0.91 |
| rs4385377 | 7 | 202 | 21661805 | C | 0.03 | 0.03 | 0.18 | 0.73 |
| rs17170516 | 7 | 283 | 32785284 | G | 0.97 | 0.74 | 0.78 | 0.15 |
| rs10244781 | 7 | 320 | 33816910 | C | 0.00 | 0.17 | 0.13 | 0.77 |
| rs12671838 | 7 | 208 | 37906849 | A | 0.16 | 0.03 | 0.04 | 0.70 |
| rs6462862 | 7 | 25 | 38738392 | A | 0.28 | 0.38 | 0.46 | 0.97 |
| rs2190336 | 7 | 277 | 41767779 | A | 0.10 | 0.17 | 0.23 | 0.78 |
| rs193869 | 7 | 209 | 87904793 | G | 0.23 | 0.22 | 0.29 | 0.84 |
| rs2523018 | 7 | 310 | 111595684 | T | 0.18 | 0.14 | 0.18 | 0.76 |
| rs13235516 | 7 | 235 | 130574221 | C | 0.13 | 0.28 | 0.39 | 0.95 |
| rs322347 | 7 | 71 | 136643724 | A | 0.05 | 0.30 | 0.41 | 0.93 |
| rs12673296 | 7 | 211 | 145778456 | G | 0.26 | 0.27 | 0.22 | 0.84 |
| rs10249351 | 7 | 26 | 145992506 | A | 0.10 | 0.21 | 0.26 | 0.96 |
| rs7804456 | 7 | 21 | 148246576 | T | 0.63 | 0.72 | 0.95 | 0.09 |
| rs1549760 | 7 | 237 | 150386772 | T | 0.20 | 0.23 | 0.29 | 0.85 |
| rs10094208 | 8 | 293 | 6169773 | C | 0.14 | 0.13 | 0.21 | 0.78 |
| rs2975659 | 8 | 173 | 10169046 | G | 0.10 | 0.15 | 0.18 | 0.76 |
| rs352806 | 8 | 15 | 15662778 | G | 0.22 | 0.24 | 0.33 | 0.97 |
| rs952859 | 8 | 48 | 20076867 | G | 0.07 | 0.25 | 0.24 | 0.82 |
| rs10954844 | 8 | 187 | 32421634 | C | 0.06 | 0.08 | 0.11 | 0.67 |
| rs1603708 | 8 | 192 | 55961290 | T | 0.18 | 0.23 | 0.25 | 0.81 |
| rs1160133 | 8 | 59 | 59467844 | T | 0.12 | 0.13 | 0.17 | 0.86 |
| rs2326457 | 8 | 240 | 61006987 | T | 0.00 | 0.05 | 0.05 | 0.74 |
| rs569688 | 8 | 10 | 61124375 | T | 0.08 | 0.19 | 0.32 | 0.99 |
| rs7835942 | 8 | 102 | 61960345 | T | 0.99 | 0.78 | 0.78 | 0.14 |
| rs1062852 | 8 | 309 | 87640368 | G | 0.18 | 0.16 | 0.37 | 0.91 |
| rs10808357 | 8 | 317 | 98664005 | T | 0.03 | 0.04 | 0.27 | 0.82 |
| rs17717583 | 8 | 122 | 116972819 | G | 0.97 | 0.93 | 0.93 | 0.39 |
| rs4077747 | 8 | 299 | 119498125 | G | 0.28 | 0.23 | 0.29 | 0.85 |
| rs4733812 | 8 | 323 | 129068822 | A | 0.00 | 0.36 | 0.18 | 0.89 |
| rs17634696 | 8 | 133 | 134291401 | C | 0.11 | 0.13 | 0.01 | 0.67 |
| rs748154 | 8 | 193 | 134777590 | A | 0.00 | 0.25 | 0.26 | 0.89 |
| rs12548628 | 8 | 87 | 139514862 | T | 0.19 | 0.42 | 0.29 | 0.93 |
| rs16931228 | 9 | 176 | 10166877 | C | 0.00 | 0.01 | 0.08 | 0.62 |
| rs10511543 | 9 | 220 | 10309468 | C | 0.01 | 0.00 | 0.06 | 0.59 |
| rs12338647 | 9 | 141 | 10442255 | C^§^ | 0.04 | 0.13 | 0.13 | 0.73 |
| rs10809140 | 9 | 32 | 10615637 | T^§^ | 0.06 | 0.39 | 0.43 | 0.95 |
| rs4741370 | 9 | 169 | 14383209 | C^§^ | 0.03 | 0.13 | 0.21 | 0.81 |
| rs1416745 | 9 | 128 | 16901091 | G^§^ | 0.17 | 0.10 | 0.16 | 0.73 |
| rs10120545 | 9 | 154 | 18376757 | T | 0.16 | 0.07 | 0.25 | 0.81 |
| rs10965507 | 9 | 294 | 22892231 | G | 0.01 | 0.34 | 0.27 | 0.88 |
| rs4246860 | 9 | 61 | 25589860 | A | 0.61 | 0.81 | 0.62 | 0.07 |
| rs12236534 | 9 | 181 | 91109688 | G | 0.13 | 0.11 | 0.28 | 0.89 |
| rs10989206 | 9 | 206 | 102472019 | A | 0.00 | 0.23 | 0.25 | 0.81 |
| rs10513270 | 9 | 186 | 118073155 | A | 0.00 | 0.12 | 0.04 | 0.68 |
| rs7096433 | 10 | 116 | 26881706 | G^§^ | 0.20 | 0.33 | 0.23 | 0.90 |
| rs867768 | 10 | 70 | 31454310 | T | 0.00 | 0.31 | 0.37 | 0.94 |
| rs7905537 | 10 | 74 | 34271281 | C | 0.04 | 0.23 | 0.27 | 0.84 |
| rs872953 | 10 | 247 | 63302608 | C | 0.00 | 0.00 | 0.09 | 0.68 |
| rs10509172 | 10 | 280 | 64273285 | T | 0.00 | 0.05 | 0.16 | 0.70 |
| rs7072702 | 10 | 18 | 89889823 | A | 0.28 | 0.46 | 0.33 | 0.97 |
| rs2185785 | 10 | 230 | 90202871 | G | 0.14 | 0.13 | 0.08 | 0.72 |
| rs1935961 | 10 | 42 | 95773232 | G | 0.25 | 0.41 | 0.44 | 0.97 |
| rs790743 | 10 | 54 | 106823886 | C | 0.23 | 0.21 | 0.18 | 0.78 |
| rs4918821 | 10 | 28 | 115190113 | T | 0.10 | 0.08 | 0.26 | 0.95 |
| rs11016304 | 10 | 222 | 130211878 | T | 0.08 | 0.25 | 0.25 | 0.83 |
| rs12243174 | 10 | 241 | 131449800 | T | 0.01 | 0.22 | 0.28 | 0.86 |
| rs7905353 | 10 | 88 | 134395400 | T^§^ | 0.17 | 0.09 | 0.28 | 0.83 |
| rs217213 | 11 | 147 | 1935694 | C | 0.73 | 0.66 | 0.66 | 0.11 |
| rs12418890 | 11 | 143 | 11849793 | A | 0.06 | 0.15 | 0.11 | 0.70 |
| rs11030679 | 11 | 218 | 29358810 | C | 0.03 | 0.13 | 0.07 | 0.68 |
| rs12576917 | 11 | 146 | 43881323 | T | 0.89 | 0.96 | 0.83 | 0.19 |
| rs11230889 | 11 | 263 | 61579962 | C | 0.11 | 0.19 | 0.16 | 0.76 |
| rs12421620 | 11 | 136 | 66033152 | A | 0.00 | 0.06 | 0.00 | 0.67 |
| rs498820 | 11 | 233 | 78741369 | C | 0.13 | 0.25 | 0.29 | 0.88 |
| rs2448269 | 11 | 249 | 79777451 | T | 0.00 | 0.17 | 0.11 | 0.78 |
| rs655484 | 11 | 127 | 83861083 | T | 0.00 | 0.00 | 0.05 | 0.62 |
| rs12419376 | 11 | 303 | 87309810 | A | 0.04 | 0.03 | 0.00 | 0.58 |
| rs1488899 | 11 | 204 | 88996075 | T | 0.00 | 0.07 | 0.21 | 0.76 |
| rs2399681 | 11 | 129 | 92931547 | G^¥^ | 0.14 | 0.02 | 0.11 | 0.85 |
| rs878874 | 11 | 295 | 94228113 | G | 0.03 | 0.16 | 0.30 | 0.91 |
| rs7925220 | 11 | 288 | 95345783 | G | 0.24 | 0.28 | 0.31 | 0.85 |
| rs2046685 | 11 | 234 | 103378701 | C^§^ | 0.05 | 0.34 | 0.32 | 0.88 |
| rs11213282 | 11 | 301 | 109507271 | G | 0.02 | 0.00 | 0.08 | 0.62 |
| rs620331 | 11 | 185 | 118436309 | T | 0.00 | 0.13 | 0.09 | 0.68 |
| rs11217793 | 11 | 19 | 119667096 | T | 0.17 | 0.19 | 0.16 | 0.92 |
| rs12418011 | 11 | 78 | 123049115 | A | 0.07 | 0.08 | 0.19 | 0.80 |
| rs713279 | 11 | 170 | 133068519 | A | 0.09 | 0.22 | 0.27 | 0.92 |
| rs11062799 | 12 | 80 | 3737524 | G | 0.02 | 0.14 | 0.20 | 0.82 |
| rs16931637 | 12 | 261 | 27676433 | G | 0.92 | 1.00 | 0.92 | 0.34 |
| rs7315895 | 12 | 43 | 28978325 | A | 0.34 | 0.39 | 0.29 | 0.93 |
| rs10506070 | 12 | 290 | 30720941 | G | 0.00 | 0.10 | 0.00 | 0.66 |
| rs4768093 | 12 | 90 | 44313582 | T | 0.09 | 0.00 | 0.09 | 0.68 |
| rs2051827 | 12 | 282 | 46242298 | A | 0.06 | 0.06 | 0.07 | 0.73 |
| rs11169212 | 12 | 44 | 48617546 | G | 0.18 | 0.02 | 0.16 | 0.76 |
| rs1921079 | 12 | 289 | 64791566 | A | 0.08 | 0.26 | 0.12 | 0.83 |
| rs10784673 | 12 | 132 | 66773727 | A | 0.87 | 0.63 | 0.62 | 0.08 |
| rs12229055 | 12 | 121 | 94852553 | A | 0.00 | 0.00 | 0.14 | 0.72 |
| rs17027872 | 12 | 266 | 97103100 | C | 1.00 | 1.00 | 0.98 | 0.49 |
| rs741543 | 12 | 145 | 107488231 | T | 0.13 | 0.08 | 0.13 | 0.67 |
| rs7967620 | 12 | 94 | 107932379 | A | 0.25 | 0.00 | 0.12 | 0.82 |
| rs34268 | 12 | 180 | 108081540 | A | 0.13 | 0.05 | 0.11 | 0.69 |
| rs12301907 | 12 | 47 | 115555362 | T | 0.08 | 0.40 | 0.44 | 0.95 |
| rs4765045 | 12 | 91 | 124557937 | A | 0.81 | 0.92 | 0.81 | 0.22 |
| rs17438276 | 12 | 178 | 124690156 | T | 0.78 | 0.86 | 0.77 | 0.19 |
| rs7955663 | 12 | 5 | 127800083 | A | 0.77 | 0.64 | 0.63 | 0.01 |
| rs12430213 | 13 | 77 | 20148738 | A | 0.00 | 0.03 | 0.00 | 0.75 |
| rs17288031 | 13 | 281 | 22578684 | G | 0.00 | 0.08 | 0.10 | 0.66 |
| rs1117380 | 13 | 279 | 27158105 | C | 0.05 | 0.30 | 0.26 | 0.91 |
| rs17626707 | 13 | 150 | 27870743 | T | 0.00 | 0.05 | 0.02 | 0.55 |
| rs2858820 | 13 | 46 | 32547629 | C | 0.08 | 0.33 | 0.23 | 0.92 |
| rs1538142 | 13 | 1 | 37344432 | C | 0.92 | 0.98 | 1.00 | 0.03 |
| rs1037272 | 13 | 259 | 41688458 | A | 0.02 | 0.12 | 0.04 | 0.69 |
| rs1023055 | 13 | 92 | 49953197 | T | 0.05 | 0.12 | 0.04 | 0.76 |
| rs1374483 | 13 | 191 | 57006356 | G | 0.11 | 0.23 | 0.25 | 0.80 |
| rs7996220 | 13 | 23 | 65813902 | T | 0.88 | 0.53 | 0.58 | 0.01 |
| rs11148861 | 13 | 11 | 70374661 | C | 0.60 | 0.64 | 0.66 | 0.04 |
| rs9573013 | 13 | 138 | 72162073 | A | 0.00 | 0.00 | 0.10 | 0.70 |
| rs9594037 | 13 | 34 | 85076105 | A | 0.07 | 0.00 | 0.00 | 0.91 |
| rs693092 | 13 | 33 | 87858156 | G | 0.81 | 0.67 | 0.56 | 0.04 |
| rs9513199 | 13 | 35 | 96443969 | T | 0.17 | 0.44 | 0.36 | 0.96 |
| rs9555408 | 13 | 175 | 107238344 | C | 0.00 | 0.12 | 0.15 | 0.80 |
| rs1571627 | 13 | 95 | 110938446 | T | 0.13 | 0.08 | 0.09 | 0.69 |
| rs10483251 | 14 | 99 | 20741117 | T | 0.08 | 0.18 | 0.07 | 0.88 |
| rs7151991 | 14 | 13 | 31705323 | A | 0.15 | 0.18 | 0.18 | 0.95 |
| rs4982052 | 14 | 45 | 32657917 | G | 0.09 | 0.33 | 0.22 | 0.93 |
| rs1954123 | 14 | 55 | 47696133 | T | 0.13 | 0.23 | 0.01 | 0.82 |
| rs4898693 | 14 | 198 | 50987244 | T | 0.03 | 0.06 | 0.06 | 0.57 |
| rs17106441 | 14 | 255 | 68406385 | C | 0.26 | 0.26 | 0.34 | 0.88 |
| rs12436698 | 14 | 223 | 70761248 | G^§^ | 0.09 | 0.09 | 0.17 | 0.77 |
| rs4453400 | 14 | 308 | 79469734 | G | 0.00 | 0.07 | 0.00 | 0.59 |
| rs9323913 | 14 | 39 | 94217063 | C | 0.80 | 0.77 | 0.67 | 0.05 |
| rs12431496 | 14 | 160 | 96829091 | G | 0.00 | 0.00 | 0.09 | 0.65 |
| rs4900438 | 14 | 73 | 99166992 | C | 0.11 | 0.18 | 0.25 | 0.81 |
| rs890319 | 15 | 297 | 24464727 | T | 0.03 | 0.34 | 0.28 | 0.88 |
| rs16950318 | 15 | 306 | 25609260 | A | 0.00 | 0.04 | 0.00 | 0.57 |
| rs11629683 | 15 | 207 | 31780435 | C^§^ | 0.00 | 0.04 | 0.02 | 0.60 |
| rs16959296 | 15 | 224 | 32731675 | C | 0.00 | 0.13 | 0.18 | 0.88 |
| rs1916306 | 15 | 4 | 35068986 | C^§^ | 0.02 | 0.08 | 0.32 | 0.97 |
| rs17545090 | 15 | 258 | 51193685 | A^¥^ | 0.00 | 0.00 | 0.00 | 0.47 |
| rs4774885 | 15 | 195 | 54732740 | G | 0.94 | 0.99 | 1.00 | 0.28 |
| rs12439270 | 15 | 52 | 58029372 | C | 0.91 | 0.68 | 0.87 | 0.07 |
| rs1425935 | 15 | 30 | 58141811 | A | 0.60 | 0.70 | 0.73 | 0.07 |
| rs1033028 | 15 | 151 | 58404227 | G | 0.22 | 0.28 | 0.33 | 0.90 |
| rs305049 | 15 | 101 | 67876287 | G | 0.25 | 0.36 | 0.34 | 0.89 |
| rs938682 | 15 | 14 | 76683602 | G | 0.27 | 0.22 | 0.49 | 0.99 |
| rs1551991 | 15 | 275 | 90695158 | T | 0.00 | 0.00 | 0.26 | 0.82 |
| rs8038709 | 15 | 56 | 91549829 | G | 0.33 | 0.26 | 0.41 | 0.93 |
| rs11248883 | 16 | 250 | 1554241 | G | 0.02 | 0.04 | 0.11 | 0.66 |
| rs4785955 | 16 | 194 | 4237652 | G | 0.78 | 0.83 | 0.82 | 0.22 |
| rs1677488 | 16 | 302 | 8861823 | G | 0.05 | 0.03 | 0.02 | 0.79 |
| rs7404672 | 16 | 190 | 10873980 | T | 0.00 | 0.01 | 0.31 | 0.88 |
| rs17605165 | 16 | 296 | 11205063 | T | 0.14 | 0.12 | 0.12 | 0.81 |
| rs12448342 | 16 | 270 | 12638459 | A^§^ | 0.03 | 0.07 | 0.10 | 0.73 |
| rs1362577 | 16 | 105 | 47416478 | G | 0.14 | 0.01 | 0.15 | 0.70 |
| rs1452501 | 16 | 130 | 79180763 | T | 0.03 | 0.07 | 0.15 | 0.81 |
| rs964453 | 16 | 164 | 83325736 | C | 0.17 | 0.20 | 0.22 | 0.78 |
| rs751048 | 16 | 174 | 84372310 | C | 0.98 | 1.00 | 0.96 | 0.38 |
| rs4790302 | 17 | 7 | 1820505 | G | 0.63 | 0.65 | 0.58 | 0.03 |
| rs12449698 | 17 | 177 | 3157666 | A | 0.00 | 0.13 | 0.22 | 0.85 |
| rs16553 | 17 | 37 | 28391976 | T | 0.58 | 0.58 | 0.55 | 0.04 |
| rs159318 | 17 | 53 | 29710751 | A | 0.05 | 0.40 | 0.45 | 0.96 |
| rs6607284 | 17 | 17 | 33100448 | C | 0.38 | 0.36 | 0.34 | 0.96 |
| rs2877398 | 17 | 159 | 48467696 | T | 0.08 | 0.09 | 0.10 | 0.63 |
| rs12948768 | 17 | 238 | 75812400 | G | 0.18 | 0.33 | 0.32 | 0.89 |
| rs680173 | 18 | 179 | 2864364 | C | 0.18 | 0.28 | 0.36 | 0.93 |
| rs17259452 | 18 | 197 | 19645032 | T | 0.07 | 0.02 | 0.06 | 0.58 |
| rs16968965 | 18 | 79 | 33312969 | C | 0.00 | 0.08 | 0.01 | 0.59 |
| rs12456856 | 18 | 251 | 47984154 | T | 0.03 | 0.17 | 0.12 | 0.72 |
| rs1498163 | 18 | 324 | 49685983 | C | 0.00 | 0.08 | 0.00 | 0.73 |
| rs1064179 | 18 | 29 | 69732579 | G | 0.35 | 0.32 | 0.24 | 0.92 |
| rs4802053 | 19 | 69 | 44839079 | A^§^ | 0.03 | 0.37 | 0.38 | 0.93 |
| rs8103085 | 19 | 201 | 56435857 | T | 0.18 | 0.02 | 0.19 | 0.74 |
| rs17249858 | 20 | 319 | 24082855 | C | 0.00 | 0.06 | 0.00 | 0.62 |
| rs12480522 | 20 | 321 | 24534383 | G | 0.11 | 0.26 | 0.04 | 0.84 |
| rs2185558 | 20 | 322 | 36339308 | C | 0.08 | 0.16 | 0.17 | 0.78 |
| rs6129317 | 20 | 239 | 37502068 | C | 0.17 | 0.41 | 0.39 | 0.94 |
| rs2866987 | 20 | 51 | 40037991 | G | 0.14 | 0.43 | 0.47 | 0.97 |
| rs6066541 | 20 | 305 | 45975466 | G^§^ | 0.22 | 0.33 | 0.16 | 0.88 |
| rs4809681 | 20 | 260 | 46261841 | C | 0.08 | 0.13 | 0.11 | 0.67 |
| rs1883886 | 20 | 119 | 46519060 | A | 0.12 | 0.14 | 0.18 | 0.73 |
| rs4999125 | 20 | 286 | 49408386 | A^§^ | 0.19 | 0.23 | 0.26 | 0.82 |
| rs17001256 | 20 | 72 | 53524033 | G | 0.03 | 0.04 | 0.08 | 0.61 |
| rs2151 | 21 | 314 | 25661883 | A | 0.11 | 0.09 | 0.15 | 0.74 |
| rs1012482 | 21 | 49 | 32104353 | G | 0.34 | 0.43 | 0.40 | 0.95 |
| rs2837352 | 21 | 38 | 40285648 | T | 0.11 | 0.17 | 0.40 | 0.96 |
| rs915837 | 21 | 298 | 42286800 | T | 0.76 | 0.71 | 0.62 | 0.09 |
| rs115525 | 22 | 126 | 23442325 | A | 0.29 | 0.25 | 0.17 | 0.88 |
| rs6005016 | 22 | 264 | 25063299 | G | 0.92 | 0.97 | 0.87 | 0.31 |
| rs470097 | 22 | 273 | 26703744 | C | 0.83 | 0.73 | 0.70 | 0.15 |
| rs2235135 | 22 | 2 | 37546613 | T | 0.38 | 0.40 | 0.28 | 0.96 |
| rs17425081 | 22 | 318 | 39145818 | A | 0.00 | 0.04 | 0.01 | 0.76 |
| rs17379759 | 22 | 157 | 40629481 | G | 0.06 | 0.11 | 0.06 | 0.68 |
| rs1557553 | 22 | 135 | 43139648 | T | 0.07 | 0.09 | 0.31 | 0.86 |
| rs6007204 | 22 | 36 | 43380054 | T | 0.08 | 0.31 | 0.40 | 0.95 |

*Ascertainment sample composed of Totonacs and non-admixed Bolivians.

^¥^Frequencies in one or more populations were substantially different from HapMap due to missing data.

^§^Original Affymetrix strand assignment is reversed relative to dbSNP for some A/T and G/C polymorphisms.

The displayed allele frequencies are standardized to dbSNP build (132).
