# Supplementary material for: Genetic analysis of ancestry, admixture and selection in Bolivian and Totonac populations of the New World
Source: BMC Genet. 2012 May 20;13:39. doi: 10.1186/1471-2156-13-39 (PMC3432609; doi:10.1186/1471-2156-13-39)
Supplement: Additional file 2: Table S2. — Highly-differentiated SNP frequencies. [file 1471-2156-13-39-S2.docx]

Supplemental Table 2. Frequency of highly-differentiated Native American SNPs in three Old World and New World samples

|  |  |  |  |  | Population frequency | | | | |
| --- | --- | --- | --- | --- | --- | --- | --- | --- | --- |
| Rs number | Chromosome | Position (hg18) | Alleles | Derived allele | YRI | CEU | CHB/JPT | Bolivians & Totonacs* | HGDP** |
| rs2320170 | 2 | 95603500 | A/G | A | 0.90 | 0.68 | 0.68 | 0.05 | 0.08 |
| rs3774089 | 3 | 10931071 | C/T^§^ | T | 0.01 | 0.11 | 0.17 | 0.87 | NA |
| rs1344869 | 3 | 21282605 | G/A^§^ | G | 0.95 | 1.00 | 0.69 | 0.07 | NA |
| rs9847307 | 3 | 64500753 | A/T | A^‡^ | 0.16 | 0.01 | 0.31 | 0.96 | 0.92 |
| rs17617120 | 5 | 155231791 | C/T | T | 0.06 | 0.12 | 0.19 | 0.89 | NA |
| rs17617422 | 5 | 155249830 | G/A | G | 0.06 | 0.12 | 0.19 | 0.95 | 0.92 |
| rs11960137 | 5 | 155270659 | G/C | G | 0.08 | 0.08 | 0.22 | 0.95 | 0.90 |
| rs2642515 | 7 | 145998474 | C/T^§^ | T | 0.90 | 0.79 | 0.74 | 0.04 | NA |
| rs174547 | 11 | 61327359 | C/T | T | 0.98 | 0.63 | 0.67 | 0.01 | 0.04 |
| rs174548 | 11 | 61327924 | G/C | C | 0.84 | 0.68 | 0.67 | 0.01 | 0.04 |
| rs174549 | 11 | 61327958 | G/A | G | 0.98 | 0.68 | 0.67 | 0.01 | 0.04 |
| rs11610143 | 12 | 50635338 | G/C^§^ | G^‡^ | 0.00 | 0.19 | 0.33 | 0.93 | 0.96 |
| rs7955663 | 12 | 127800083 | A/T | A | 0.77 | 0.64 | 0.63 | 0.01 | 0.02 |
| rs1538142 | 13 | 37344432 | C/T^§^ | C | 0.92 | 0.98 | 1.00 | 0.03 | NA |
| rs693092 | 13 | 87858156 | G/A | G | 0.81 | 0.67 | 0.56 | 0.04 | 0.10 |
| rs9515075 | 13 | 88033482 | C/T | C | 0.80 | 0.63 | 0.61 | 0.01 | 0.04 |
| rs566514 | 13 | 32551339 | C/T^§^ | T | 0.98 | 0.68 | 0.76 | 0.07 | 0.08 |
| rs7170342 | 15 | 32755246 | C/T | C | 0.82 | 0.62 | 0.54 | 0.01 | NA |
| rs4924116 | 15 | 35086443 | C/T | C | 0.99 | 0.92 | 0.63 | 0.03 | 0.04 |
| rs12439270 | 15 | 58029372 | C/A | C | 0.91 | 0.68 | 0.87 | 0.07 | 0.14 |
| rs1452501 | 16 | 79180763 | C/T | T | 0.03 | 0.07 | 0.15 | 0.81 | 0.80 |
| rs470113 | 22 | 39059560 | G/A | A | 0.90 | 0.80 | 0.68 | 0.08 | 0.14 |

*Non-admixed Bolivians and Totonacs; **pooled HGDP samples consisting of Pima (5), Maya (5), Colombian (5), Karitiana (5), and Surui (5); ^‡^rs9847307 and rs11610143 standardized to dbSNP build (132); ^§^UCSC plus strand; NA: not available
