# Supplementary material for: Genetic analysis of ancestry, admixture and selection in Bolivian and Totonac populations of the New World
Source: BMC Genet. 2012 May 20;13:39. doi: 10.1186/1471-2156-13-39 (PMC3432609; doi:10.1186/1471-2156-13-39)
Supplement: Additional file 3: Table S3. — Haplotypes and haplotype frequencies associated with the highly-differentiated SNPs. Genotype data and Affymetrix cel files for the Totonac and Bolivian samples can be downloaded from the Gene Expression Omnibus (GEO) archive (GSE29851). [file 1471-2156-13-39-S3.docx]

Supplemental Table 3. Frequency of highly-differentiated haplotypes in native Bolivians, Totonacs and pooled New World HGDP samples.

| Identifying  SNP | Chr | Gene | Core haplotype  (merged data) | Start SNP | Start position | End SNP | End position | Size  (kb) | Haplotype frequency | |
| --- | --- | --- | --- | --- | --- | --- | --- | --- | --- | --- |
|  |  |  |  |  |  |  |  |  | Bolivians & Totonacs | HGDP |
| **rs2320170** | 2 | 5' of TRIM43 | CCCCGAAGCGGCCT**G**G | rs2320625 | 95226463 | rs4907276 | 95765853 | 539.4 | 0.68 | 0.68 |
| **rs3774089** | 3 | SLC6A11 | ACGGTCGCTGCGTC**\|**CTATGCAGTAG | rs11918616 | 10884566 | rs2697160 | 10950251 | 65.7 | 0.81 | 0.78 |
| **rs1344869** | 3 | - | CGTTGGATCGACTTA**\|**CTTGCAAAGTG | rs1898300 | 21216660 | rs17008485 | 21365199 | 148.5 | 0.91 | 0.86 |
| **rs9847307** | 3 | ADAMTS9 | CCTTTTGCCC | rs1015859 | 64493161 | rs9311896 | 64514815 | 21.7 | 0.76 | 0.64 |
| **rs17617120** | 5 | SGCD | AGAT**AG**GAATTC**G**TCCTA | rs7378774 | 155221392 | rs6894361 | 155304317 | 82.9 | 0.92 | 0.86 |
| **rs17617422** | 5 | SGCD | See rs17617120 |  |  |  |  |  |  |  |
| **rs11960137** | 5 | SGCD | See rs17617120 |  |  |  |  |  |  |  |
| **rs2642515** | 7 | CNTNAP2 | A**\|**AAAGCACAAATACGGTT | rs10249351 | 145992506 | rs344470 | 146044430 | 51.9 | 0.96 | 0.78 |
| **rs174547** | 11 | FADS1 | GTA**CGA**T | rs174534 | 61306034 | rs174570 | 61353788 | 47.8 | 0.97 | 0.96 |
| **rs174548** | 11 | FADS1 | See rs174547 |  |  |  |  |  |  |  |
| **rs174549** | 11 | FADS1 | See rs174547 |  |  |  |  |  |  |  |
| **rs11610143** | 12 | ACVR1B | TTGGATGACA**G**ACAGA | rs11169954 | 50598555 | rs12830373 | 50663263 | 64.7 | 0.92 | 0.94 |
| **rs7955663** | 12 | - | **A**TGG | rs1077410 | 127797029 | rs11059871 | 127800764 | 3.7 | 0.97 | 0.98 |
| **rs1538142** | 13 | 5' of TRPC4 | CG**\|**TGC | rs1538146 | 37343288 | rs17056770 | 37350095 | 6.8 | 0.92 | 0.75 |
| **rs693092** | 13 | - | GTACGTATAT**A**TAAGCTCCGCCTTCCTTGCTGACTTATT**T**TCCGGTGT | rs9520372 | 87812863 | rs4773045 | 88057701 | 244.8 | 0.91 | 0.88 |
| **rs9515075** | 13 | - | See rs693092 |  |  |  |  |  |  |  |
| **rs566514** | 13 | 5' of STARD13 | GT**C**AG | rs17763040 | 32543384 | rs7988511 | 32560185 | 16.8 | 0.93 | 0.92 |
| **rs7170342** | 15 | - | TCGAA**\|**T | rs11073055 | 32751250 | rs6495705 | 32757729 | 6.5 | 0.97 | 0.92 |
| **rs4924116** | 15 | MEIS2 | ATTCGCCT**T**CCT | rs8034391 | 35059564 | rs11852575 | 35115441 | 55.9 | 0.77 | 0.68 |
| **rs12439270** | 15 | 5'of FOXB1 | **A**TTGC | rs12439270 | 58029372 | rs2970378 | 58049895 | 20.5 | 0.93 | 0.86 |
| **rs1452501** | 16 | - | GCA**T**TTC | rs1466183 | 79175194 | rs10395 | 79189791 | 14.6 | 0.78 | 0.80 |
| **rs470113** | 22 | TNRC6B | **G**AGTACAAGAACGTTGCCCTGCGTTGTGAACTTGCTCTGCTTCCATGCGAGTTGAGATG | rs470113 | 39017703 | rs738633 | 39768656 | 751.0 | 0.72 | 0.70 |

Identifying SNP positions are underlined; Chr: chromosome; **|** shows the location of the SNP in the haplotype if the highly-differentiated SNP was not present in the merged data set; positions based on hg18.
